# Psychopathic traits modulate functional connectivity during pain perception and perspective-taking in female inmates

**DOI:** 10.1016/j.nicl.2022.102984

**Published:** 2022-03-05

**Authors:** Keith J. Yoder, Carla L. Harenski, Kent A. Kiehl, Jean Decety

**Affiliations:** a Department of Psychology, University of Chicago, Chicago, IL, United States; b The Mind Research Network and Lovelace Biomedical, Albuquerque, NM, United States; c Department of Psychology, University of New Mexico, Albuquerque, NM, United States; d Department of Psychiatry and Behavioral Neuroscience, University of Chicago, Chicago, IL, United States

**Keywords:** Empathy, Perspective-taking, Psychopathy, Functional connectivity, Functional MRI, Female psychopathy

## Abstract

The ability to share and understand the distress of others is critical for successful social interactions and is a fundamental building block of morality. Psychopathy is a personality disorder that includes lack of empathy and concern for others. In the present study, functional MRI was used to examine neural responses and functional connectivity associated with empathy and affective perspective-taking in female inmates (N = 109) with various levels of psychopathic traits, as measured with Psychopathy Checklist-Revised (PCL-R). Participants viewed hands and feet in painful or non-painful situations while adopting a first person or third person perspective. All participants demonstrated robust neural responses in anterior insula (aINS), anterior cingulate (ACC), temporoparietal junction (TPJ) and supplementary motor area (SMA) when viewing pain, both during imagine-self and imagine-other blocks. Psychopathy shifted the functional connectivity seeded in core nodes of the salience and social cognition networks. Perceiving stimuli depicting somatic pain led to decreased functional coupling from right temporoparietal junction to superior temporal sulcus, which correlated with scores on PCL-R Factor 1 (Affective/Interpersonal). In contrast, connectivity from right insula to precuneus increased with Factor 2 (Lifestyle/Antisocial) scores. When adopting a third-person perspective, psychopathic traits modulated connectivity from the social cognition network, but not the salience network, with Factor 1 scores associated with increased connectivity to sensorimotor cortex and temporal pole, while Factor 2 scores were associated with decreased connectivity with ACC/SMA and inferior frontal gyrus. Overall, these results demonstrate that psychopathic traits in incarcerated females are associated with atypical functional connectivity within the salience network during pain-empathy processing and within the social cognition network during affective perspective-taking.

## Highlights

• PCL-R scores are associated with altered functional connectivity in female inmates.
• PCL-R Factor 1 and Factor 2 subscores predicted opposite shifts in connectivity.
• Functional connectivity in the salience network is altered during pain perception.
• Connectivity in the social cognition network is altered during perspective-taking.

## 1 Introduction

Empathy is an integral part of our humanity. Indeed, empathy plays a vital role in our interpersonal life, from bonding between parents and child, to enhancing affiliation among conspecifics, to understanding others’ feelings and subjective psychological states. Empathy reflects an innate ability to perceive and be sensitive to the emotional states of others, coupled with a motivation to care for their wellbeing. This construct includes affective, motivational, and cognitive components (Decety and Jackson, 2004). These components interact but are partly dissociable in terms of neurobiological mechanisms and functions. Affective empathy, or vicariously experiencing someone else’s emotions (in valence and intensity), plays a role in motivating prosocial behaviors in both non-human animals (e.g., Ben-Ami Bartal et al., 2016) and humans (e.g., Tomova et al., 2017). An aversion to the distress of others is a crucial building block of morality (Decety and Cowell, 2018). Deficits in empathy, such as those observed in individuals with psychopathy, produce serious personal, interpersonal, and societal consequences. Despite a prevalence of around 1% in the general population (Hare, 2003, Neumann and Hare, 2008), the incidence of psychopathy among incarcerated populations may be as high as 25% (Neumann et al., 2015, Verschuere et al., 2018). Moreover, the estimated cost of psychopathy in the United States is nearly half a trillion dollars each year, making it the most expensive mental health disorder (Kiehl and Hoffman, 2011).

Psychopathy is a personality disorder that manifests as a syndrome characterized by a constellation of affective, interpersonal, lifestyle, and antisocial features. These traits can be mapped onto two higher order factors. The affective and interpersonal aspects are collectively termed Factor 1, while the remaining features comprise Factor 2. In terms of socioemotional processing, individuals with psychopathy are characterized as being callous, lacking empathy, guilt, or remorse, and demonstrating deficient and shallow affect (Hare and Neumann, 2008). Interpersonally, individuals with high levels of psychopathic traits are arrogant, grandiose, deceitful, and manipulative (De Brito et al., 2021). Thus, reduced empathic responding is a critical component of psychopathy and uniquely distinguishes this disorder from other related disorders such as addiction or ADHD (Blair, 2005, De Brito et al., 2021). Moreover, psychopathy is reliably associated with atypical neural responses during a variety of socioemotional tasks, including the perception of pain in others (Birbaumer et al., 2005, Decety et al., 2013a, Harenski et al., 2010, Poeppl et al., 2019, Yoder et al., 2015a). Across these tasks, individuals with high levels of psychopathic traits show reduced hemodynamic responses and functional connectivity at core nodes of neural networks underlying social cognition (Deming and Koenigs, 2020, Yoder and Decety, 2018).

In healthy adults, the amygdala, dorsal anterior cingulate (dACC), anterior insula (aINS), striatum, and ventromedial prefrontal cortex (vmPFC) interact to update and maintain stimulus-value mappings and direct other cortical networks to respond to personally relevant internal and external stimuli (Chiong et al., 2013, Decety and Yoder, 2017). In particular, nociception is critical for survival and promotes the organism’s health and integrity. Nociceptive stimuli reliably elicit increased signal in dACC and aINS (Tanasescu et al., 2016), likely because these signals are salient (Legrain et al., 2011), though some have argued that dACC is selective for pain (Lieberman and Eisenberger, 2015). A large body of evidence from social neuroscience studies suggest that empathy largely relies upon simulating other's emotions grounded in shared neural representations that are engaged during first-hand emotional experiences (Decety, 2011, Lamm et al., 2011, Yamada and Decety, 2009). Developmentally, affective and motivational empathy develop earlier than cognitive empathy (also called perspective taking). These early empathic responses depend on bottom-up processes supported by the aINS, vmPFC, amygdala, and hypothalamus (Decety and Holvoet, 2021, Decety and Svetlova, 2012). Functional magnetic resonance imaging (fMRI) studies have consistently found a large overlap between clusters in the anterior cingulate cortex (ACC) and anterior insula associated with the first-hand experience of pain and the vicarious experience of pain (Fallon et al., 2020, Fan et al., 2011, Lamm et al., 2011, Lockwood, 2016, Shackman et al., 2011). In fact, classifiers trained to distinguish noxious experiences (e.g., pain, disgust) using voxels in aINS and dACC can classify vicarious pain and discomfort from a conspecific (Corradi-Dell’Acqua et al., 2016; but see Zhou et al., 2020).

However, social competence also requires cognitive abilities to adopt the perspective of another person. Perspective-taking can create feelings of closeness and further motivate prosocial behaviors. This construct largely overlaps with theory of mind, or the capacity to understand the mental states (intentions, desires, beliefs, emotions) of oneself and others. Theory of mind relies on an interconnected network of regions including the temporoparietal junction (TPJ), precuneus and posterior cingulate cortex (PCC), and mPFC/ACC (Rameson et al., 2012, Ruby and Decety, 2004, Schurz et al., 2021, Schurz et al., 2014, Young and Saxe, 2009). The right TPJ is causally involved in embodied perspective-taking and social cognition that involves imagining the self in the place of another person (Jackson et al., 2006, Martin et al., 2020, Ruby and Decety, 2004, Ruby and Decety, 2001). Meta-analytic evidence suggests that the left TPJ plays a pivotal role in both mental state understanding and visual perspective-taking (Carter and Huettel, 2013, Decety and Lamm, 2007, Schurz et al., 2013). Moreover, the right TPJ (rTPJ) is particularly critical for spontaneous mental state attributions (Boccadoro et al., 2019). Thus, in addition to aINS and dACC, right and left TPJ are important nodes to interrogate when investigating psychopathy, since psychopaths often have the capacity to understand others, but fail to do so unless motivated by some external reason such as task demands (Anderson et al., 2017, Cima et al., 2010, Lockwood, 2016, Yoder et al., 2015a).

Early work demonstrated reduced skin conductance response to the first-hand experience of pain in individuals with psychopathic traits (Birbaumer et al., 2005), as well as a blunted response to signals of others’ distress (Aniskiewicz, 1979, Blair et al., 1997). However, results from fMRI studies have been more nuanced and somewhat inconclusive. In a subclinical sample, higher Factor 1 and Factor 2 traits were associated with reduced responses in the amygdala and aINS when perceiving other people’s emotional facial expressions (Seara-Cardoso et al., 2016). Similarly, inmates who scored high on the PCL-R showed a reduction in neuro-hemodynamic response to facial expressions of fear, sadness, happiness and pain in the face processing network (inferior occipital gyrus, fusiform gyrus, and superior temporal sulcus [STS]) as well as the extended network (inferior frontal gyrus [IFG] and orbitofrontal cortex [OFC]) (Decety et al., 2014). However, the hemodynamic response in aINS when perceiving facial expressions of pain, fear, and sadness was positively correlated with scores on both Factors 1 and 2, as indexed by the Psychopathy Checklist Revised (PCL-R).

When perceiving videos of hands inflicting harm or caressing, individuals with psychopathy demonstrated reduced hemodynamic response in dACC and aINS. This difference was reduced by instructing participants to empathize with the actors’ hands (Meffert et al., 2013). Similarly, when incarcerated males were asked to evaluate the moral status of harmful compared to helpful interactions, higher levels of psychopathic traits were associated with reduced hemodynamic response in nodes of the salience and social cognition networks, specifically, TPJ, dACC, and amygdala (Yoder et al., 2015a). However, when explicitly asked to attend to individuals experiencing somatic pain, higher psychopathic traits were instead associated with increased hemodynamic response in dACC and aINS (Decety et al., 2013b). Psychopathy scores also predict increased signal in dACC and aINS when incarcerated males are asked to adopt a first-person perspective when viewing a hand or foot in pain (Decety et al., 2013a). Recent *meta*-analytic evidence suggests that across a range of experimental paradigms psychopathy is associated with increased responses in fronto-insular cortex and decreased response in amygdala (Poeppl et al., 2019). Thus, it appears that sensorimotor processing of pain seems to not be absent in psychopathy (Decety et al., 2013a), but that this signal does not influence downstream stimulus-outcome mappings (Moul et al., 2012).

This disruption of information transmission in psychopathy also manifests as alterations in structural and functional neural networks. For instance, several studies have found that psychopathy is associated with reduced white matter integrity in the uncinate fasciculus, the tract which connects the amygdala and aINS with vmPFC and the rest of the inferior frontal cortex (Motzkin et al., 2011, Wolf et al., 2015). Several large-scale investigations have also linked higher psychopathy traits to altered resting state connectivity, such as reduced functional connectivity between attentional, default, and salience networks (Espinoza et al., 2018) or fewer hubs in subcortical regions, like the amygdala, alongside increased efficiency within default and dorsal attention networks (Tillem et al., 2019). This disrupted network architecture leads to atypically functional connectivity during a wide range of tasks. Indeed, higher levels of psychopathic traits in adults, as well as children with callous and unemotional traits, are reliably associated with reduced functional connectivity between core nodes of salience and social cognition networks during empathy tasks (Decety et al., 2013a, Marsh et al., 2013, Marsh et al., 2011, Passamonti et al., 2012, Waller et al., 2019, Yoder et al., 2016, Yoder et al., 2015a).

Importantly, the vast majority of cognitive neuroscience and functional neuroimaging evidence related to altered neural functioning in psychopathy comes from incarcerated males. This is not entirely surprising since incarcerated males appear to be about twice as likely to demonstrate high levels of psychopathic traits as incarcerated females (De Brito et al., 2021). However, empirical evidence is critical to clarify the impact of psychopathic traits on socioemotional processing in female inmates. Some studies have reported gender differences in empathy (e.g., Hall and Matsumoto, 2004, but see Löffler and Greitemeyer, 2021, Michalska et al., 2013). In particular, healthy female participants demonstrate greater hemodynamic response in somatomotor cortex compared to male counterparts when perceiving somatic pain (Christov-Moore and Iacoboni, 2019). Thus, the impact of psychopathic traits on pain processing in females may be reduced, and that may lessen their empathy deficits. Moreover, some work suggests that there are important differences in how psychopathy manifests in males and females. Large-scale functional connectivity analyses in incarcerated males highlights Factor 1 as a driving force behind altered network connectivity (Espinoza et al., 2018, Thijssen and Kiehl, 2017). However, while women do demonstrate an association between interpersonal PCL-R subscores and reduced uncinate fasciculus integrity (Lindner et al., 2017), Factor 2 is uniquely associated with functional connectome alterations in right insula and SMA-vmPFC connectivity. Similarly, both male and female inmates demonstrate reduced connectivity between dACC and right amygdala when evaluating harmful compared to helpful interactions, but this effect is driven by Factor 1 in males and Factor 2 in females (Yoder et al., 2021, Yoder et al., 2015a).

To examine empathic processing in females with varying levels of psychopathic traits, the current study used a validated approach that measures the hemodynamic responses to the perception of pain in others as well as affective perspective-taking (Decety et al., 2013a, Jackson et al., 2006). The impact of psychopathic traits on whole-brain responses was expected to be minimal, and instead manifest as altered network connectivity in specific networks. Core nodes of the salience network (dACC, aINS) and social cognition network (TPJ, amygdala) were selected as seeds most likely to be impacted during pain processing or perspective-taking, respectively. Based on one previous investigation of socioemotional processing in female inmates (Yoder et al., 2021), Factor 2 scores were expected to predict decreased functional connectivity with regions in the dorsal attention, salience, and default networks, while Factor 1 scores were expected to show the reverse pattern.

## 2 Methods

### 2.1 Participants

A total of 120 women completed all aspects of the study protocol. All women were incarcerated in a medium-maximum security state prison. 11 women were removed from the data analysis for excessive movement during MRI scanning (translation > 3 mm or rotation > 3 degrees), leaving a final sample of 109 (M_age_ = 35.0, SD = 8.1, range = 20–53. All right-handed). Inclusion criteria were female sex (not transitioning), age 18–59 years, not currently pregnant, reading level of at least 5th grade, ability to speak and understand English, no uncorrectable auditory or visual deficits, no lifetime history of psychotic disorder, no self-reported psychotic disorder with psychiatric hospitalization in first degree relative, no drug use in the last three months (self-report or institutional records), no central nervous system disease, no current major medical conditions, and no hypertension with complications. The women were compensated at a rate proportional with institutional wages of the correctional facility for work assignments. All materials and procedures, including recruitment, consent, and compensation within the correctional facility, were approved by the Institutional Review Boards at the University of Chicago and Ethical and Independent Review Services.

Trained research assistants administered the Hare Psychopathy Checklist-Revised (PCL-R; Hare, 2003). The PCL-R assesses four correlated facets which map onto two higher order factors (Hare, 2016). Factor 1 captures affective and interpersonal aspects, while Factor 2 captures antisocial, developmental, and lifestyle dimensions of psychopathy. Intelligence Quotient (IQ) was assessed using the matrix reasoning and vocabulary subsets from the Wechsler Abbreviated Scale of Intelligence 2nd Edition or the Wechsler Adult Intelligence Scale 3rd Edition.

### 2.2 Affective perspective-taking task

**Fig. 1:**
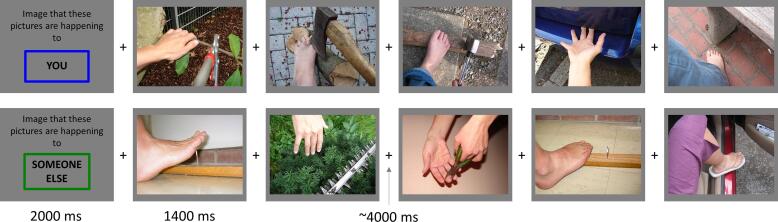
**Task Schematic**. Sample blocks for imagine-self (top) and imagine-other (bottom) blocks.

Participants completed an affective perspective-taking task that has been previously used with incarcerated males (Decety et al., 2013a) and community samples (Jackson et al., 2006). The stimuli depicted hands and feet in painful or non-painful situations (Fig. 1). Pre-scan instructions asked participants to either imagine experiencing the feeling of the hand or foot, or imagine watching another person experiencing the situation. In the scanner, participants viewed photographs in blocks of five. At the start of each block, they were cued to imagine that the hand or foot was their own or that of another person. Photographs were separated by a jittered fixation cross (M = 4 s, SD = 1 s). Blocks were separated by a 10 s rest fixation. Participants were shown 12 blocks of each type, divided over two scanning runs.

### 2.3 MRI acquisition

Functional images were acquired with the Mind Research Network 1.5 Tesla Siemens Magnetom Avanto Mobile unit (Washington, DC, USA) using a 32-channel head coil. A multiband echo-planar sequence was used (posterior-to-anterior phase encoding, multiband factor = 12, repetition time = 350 ms, echo time = 39 ms, flip angle = 37 degrees, field of view = 248 × 248 mm, matrix = 70 × 70, voxel size = 3.5 × 3.5 × 3.5 mm^3^). Images were preprocessed and analyzed using SPM12 (Wellcome Department of Imaging Neuroscience, London, UK) in MATLAB (Mathworks, Natick, MA, USA). EPI images were realigned and motion-corrected using the INRIAlign toolbox (Freire et al., 2002). Slice-timing correction was omitted in favor of using derivate boosting for first-level contrasts (Calhoun et al., 2004). EPI images were normalized directly to the MNI EPI template (Calhoun et al., 2017) and smoothed with an 8 mm Gaussian kernel.

### 2.4 Functional activation analysis

General linear modeling (GLM) estimated first level contrasts by convolving the canonical hemodynamic response function with a boxcar for the onsets and durations of each stimuli. Separate regressors were used for the four conditions: self-pain, self-nopain, other-pain, other-nopain. The temporal derivative was included, and the beta images for each temporal derivative and magnitude were combined to obtain a single magnitude estimate for use at the second-level. The six movement parameters were also included as nuisance regressors. Second-level statistical images were thresholded using height p <.001 with cluster extent of 60, corresponding to familywise error of p <.05 as determined using 3dClustSim with smoothness estimated from first-level residuals using 3dFWHMx in AFNI (version 17.2.06).

### 2.5 Functional connectivity analysis

Functional connectivity was assessed by using a generalized psychophysiological interaction (PPI) framework as implemented in the gPPI toolbox (McLaren et al., 2012). Whereas standard PPI (sPPI) computes the interaction between a deconvolved neural estimate and a single task contrast, gPPI estimates distinct PPI regressors for each task condition (Cisler et al., 2014). In both simulated and real fMRI datasets, contrasting these beta weights with gPPI provides more accurate estimates of task-related functional connectivity than sPPI (McLaren et al., 2012).

Functional coupling was examined between perspective-taking blocks using seeds placed in dACC and bilateral aINS, amygdala, and rTPJ. These regions were selected based on previous work implicating them in vicarious pain and salience processing, socioemotional processing, and disruptions in psychopathy (Bzdok et al., 2012, Deming and Koenigs, 2020, Legrain et al., 2011, Yoder and Decety, 2018). Region of interest (ROI) masks for right and left amygdala were defined anatomically using the automated anatomical atlas (AAL). Exact anatomical boundaries for aINS are ill-defined, so aINS ROIs were created using Neurosynth with the search term “anterior insula” then thresholded at z = 10. For the TPJ and dACC seeds, masks were created using a 10 mm radius sphere placed according to previous literature investigating perspective-taking, empathy for pain, and social decision-making. Specifically, coordinates from left TPJ (x = -41, y = -59, z = 42) were taken from a *meta*-analysis of false-belief and visual perspective taking (Schurz et al., 2013). Coordinates for dACC (x = -2, y = 20, z = 28) and rTPJ (x = 52, y = -54, z = 16) were taken from a *meta*-analysis of empathy, theory of mind, and morality (Bzdok et al., 2012), and have been previously linked to disrupted connectivity in psychopathy (Yoder et al., 2015a). Psychopathic traits were modeled by entering Factor 1 and Factor 2 scores at the second-level, controlling for age and IQ. Global signal was not included in models (Saad et al., 2012).

## 3 Results

### 3.1 Sample characteristics

The current sample included 27 (24.8%) women whose PCL-R total scores met or exceeded the traditional cutoff of 30 (Hare, 2016), and these women thus exhibited corresponding high Factor 1 (M = 13.3, SD = 1.9) and Factor 2 (M = 16.4, SD = 2.0) scores. Descriptive statistics, including Spearman correlations, are shown in Table 1. PCL-R scores were not significantly related to conviction for at least one violence crime (odds ratio = 1.02, p = 0.350).

**Table 1:** Descriptive statistics and bivariate Spearman correlations.

	1	2	3	4	5
1 Age	–				
2 IQ	0.07	–			
3 PCL-R	−0.19*	−0.22*	–		
4 Factor 1	−0.18	−0.22*	0.82*	–	
5 Factor 2	−0.12	−0.18	0.89*	0.52*	–
Mean	35.07	95.65	22.91	8.87	11.91
SD	8.11	12.06	7.95	3.93	4.40

PCL-R: Psychopathy Checklist-Revised. *p <.05.

### 3.2 Whole-brain results

Perceiving stimuli depicting somatic pain, compared to no-pain stimuli, was associated with greater hemodynamic response in several clusters across the “pain matrix” (Legrain et al., 2011), including a cluster in right IFG extending into aINS, amygdala, and striatum, as well as a large cluster in the frontal midline extending through the dorsal and medial PFC into SMA and dACC (Table S1, Figure S1). Conversely, several cortical regions showed greater response for the no-pain condition, including bilateral occipital and parietal cortices, right superior temporal gyrus, and left precentral gyrus.

**Fig. 2:**
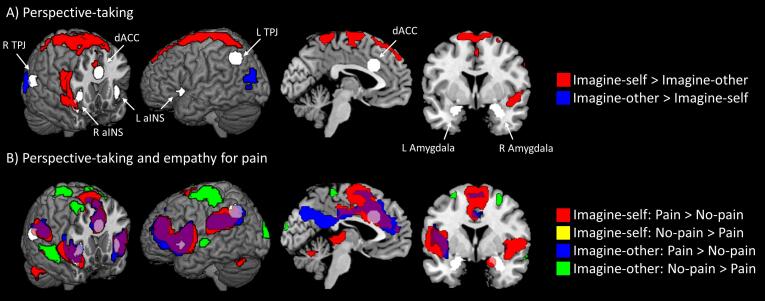
**Whole-brain results for perspective-taking and empathy for pain**. A) Regions showing greater response for imagine-self (red) or imagine-other (blue) blocks. B) Regions with greater response to pain during imagine-self (red), imagine-other (blue) or both (purple), and regions with greater response for no-pain during imagine-other blocks (green). All clusters significant at family-wise error < 0.05 (height p =.001, extent k = 60). A priori regions of interest used as seeds in the functional connectivity analysis are shown in white. dACC: dorsal anterior cingulate cortex; R aINS: right anterior insula cortex; L aINS: left anterior insula cortex; R TPJ: right temporoparietal junction; L TPJ: left temporoparietal junction. (For interpretation of the references to colour in this figure legend, the reader is referred to the web version of this article.)

The main effect of perspective-taking revealed greater response in the extrastriate body area and right fusiform gyrus for the imagine-other condition. In contrast, imagine-self blocks were associated with greater signal increase in right insula and a large midline cluster extending from SMA and dACC to somatosensory cortex (Figure 2 and S1, Table S1).

When comparing pain to no-pain stimuli within the imagine-self condition or within the imagine-other condition, many of the same regions were identified (Fig. 2, Table S2). There were overlapping clusters in bilateral TPJ, aINS, dACC, and SMA, along with left dlPFC. Pain responses during imagine-self blocks were associated with additional activation in middle temporal sulcus, thalamus, and bilateral cerebellum. Conversely, imagine-other blocks demonstrated additional activation in rostral and ventral mPFC, as well as precuneus. For both perspectives, non-painful stimuli were associated with increased response in right dlPFC. The No-pain > Pain contrast revealed increased response in right superior parietal and lateral occipital during imagine-self blocks. Conversely, during imagine-other blocks non-painful stimuli were associated with increased hemodynamic response in somatosensory cortex, superior temporal sulcus, and middle temporal sulcus. Differences in sensitivity to pain between perspective blocks were assessed with an interaction (Table S1), which revealed significantly greater response to pain in right insula during imagine-self than during imagine-other blocks.

At the whole-brain level for hemodynamic contrasts, there were no significant clusters in any of the contrasts which were significantly associated with PCL-R scores.

### 3.3 Functional connectivity during pain perception

**Fig. 3:**
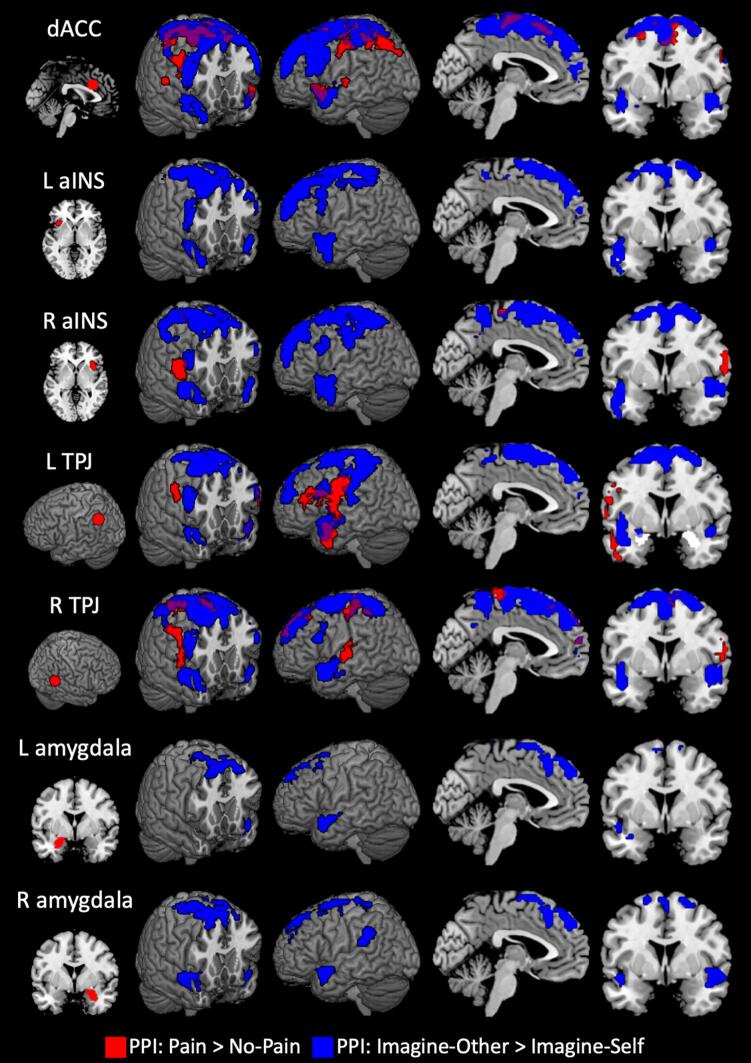
**Functional connectivity results**. Significant clusters for the psychophysiological interactions (PPI) during pain perception (red) or perspective-taking (blue). Seeds are shown at left. All clusters significant at family-wise error < 0.05 (height p =.001, extent k = 60). dACC: dorsal anterior cingulate cortex; aINS: anterior insula cortex; TPJ: temporoparietal junction. (For interpretation of the references to colour in this figure legend, the reader is referred to the web version of this article.)

Regions demonstrating significant functional connectivity with the *a priori* seeds during either task are shown in Fig. 3. When viewing painful compared to non-painful stimuli, dACC demonstrated significantly increased connectivity with somatosensory cortex, including precentral gyri, paracentral lobule, SMA, as well as bilateral posterior insula and cuneus (Figure 3 and S2, Table S3). The seeds in right aINS and right TPJ showed greater connectivity with precentral and paracentral cortices. Increased functional connectivity with rTPJ was additionally detected in SMA and rostral mPFC. Left TPJ showed increased connectivity with right postcentral gyrus and a left postcentral cluster that extended into IFG and temporal pole. No clusters showed significantly increased connectivity seed from left aINS or bilateral amygdala, and no seeds demonstrated significantly decreased connectivity with any region.

**Fig. 4:**
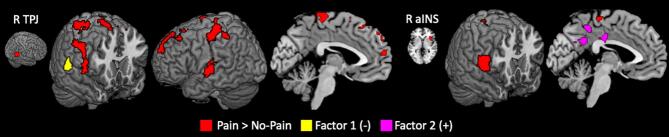
**Psychopathy and functional connectivity during pain perception**. A priori seeds and significant clusters for the psychophysiological interaction comparing painful stimuli to non-painful stimuli. Regions where connectivity was negatively related to Factor 1 scores (yellow) and regions positively related to Factor 2 (magenta). All clusters significant at family-wise error < 0.05 (height p =.001, extent k = 60). R TPJ: right temporoparietal junction; R aINS: right anterior insula. (For interpretation of the references to colour in this figure legend, the reader is referred to the web version of this article.)

Psychopathy factors scores were significantly related to alterations in functional connectivity during pain perception (Fig. 4, Table S4). Factor 1 scores were negatively associated with rTPJ-seeded connectivity and right posterior temporal sulcus. Conversely, there was a positive association between Factor 2 scores and a cluster in posterior cingulate and precuneus. No other region demonstrated significant associations between psychopathy scores and pain-related functional connectivity for any of the ROIs.

### 3.4 Functional connectivity during perspective-taking

**Fig. 5:**
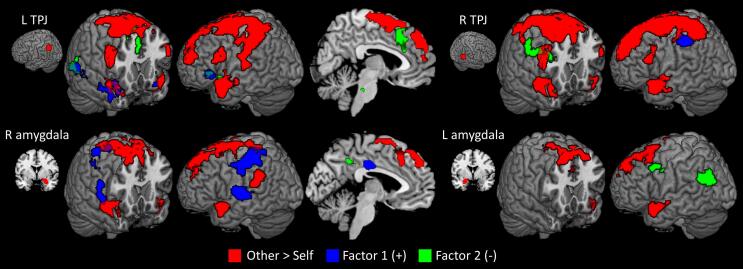
**Perspective-taking and function connectivity in the social cognition network**. Significant clusters for the psychophysiological interaction comparing imagine-other and imagine-self blocks seeded in nodes of the social cognition network. Regions where connectivity was positively related to Factor 1 scores on the PCL-R are shown in blue, and regions negatively related to Factor 2 are shown in green. All clusters significant at family-wise error < 0.05 (height p =.001, extent k = 60). R TPJ; right temporoparietal junction; L TPJ: left TPJ. (For interpretation of the references to colour in this figure legend, the reader is referred to the web version of this article.)

The functional connectivity analysis identified a common network centered in SMA and somatosensory cortex which demonstrated greater connectivity with each of the *a priori* ROIs during imagine-other than imagine-self blocks (Fig. 3, Table S5). Whether using seeds from the salience network (dACC, bilateral aINS) or social cognition network (Fig. 5), connectivity was also increased with bilateral dlPFC and temporal pole, except for the left amygdala seed which did not show greater connectivity with left temporal pole. Additionally, right amygdala showed increased connectivity with left TPJ.

Psychopathy scores were not significantly related to connectivity seeded in dACC, left aINS, or right aINS (raINS). However, PCL-R Factor scores were associated with shifts in connectivity seeded in the social cognition ROIs of TPJ and amygdala (Fig. 5, Table S6). Factor 1 scores predicted increased connectivity from left TPJ to bilateral temporal pole, left pSTS, cuneus, precuneus, and left insula. There was also a positive association between Factor 1 scores and connectivity was from right TPJ to left postcentral gyrus and from right amygdala to somatosensory cortex, left superior temporal sulcus, the right operculum, left TPJ, and midcingulate cortex.

Factor 2 scores were associated with reduced connectivity from left TPJ to left pSTS, dACC/SMA, left anterior and posterior insula, and midbrain. Connectivity seeded in right TPJ was negatively associated with Factor 2 scores in right dlPFC, as was connectivity seeded in right amygdala to posterior cingulate, and left amygdala to left TPJ, precuneus, and left dlPFC. A post-hoc analysis including the four facets of the PCL-R and using a more liberal threshold (p =.005, k = 10), revealed that the effects in this study are related more to the affective aspect of Factor 1 and the antisocial aspect of Factor 2.

## 4 Discussion

The ability to vicariously experience the emotions of other people is a fundamental aspect of social cognition (Lockwood, 2016) and represents a core mechanism of empathy. Furthermore, detecting and responding to the pain and distress cues of others is critical for normal moral development (Decety and Cowell, 2018). The current study investigated the impact of psychopathic traits on the neural underpinnings of empathy and affective perspective-taking in a large sample of incarcerated females, a population much less studied than their male counterparts.

As predicted, psychopathy scores were not significantly associated with alterations in whole-brain activations in female inmates. Instead, psychopathy factor scores were associated with distinct shifts in functional connectivity seeded in the core nodes of salience and social cognition networks, especially for the social nodes during perspective-taking (see Table S7 for a summary of regions and their potential functional significance). Taken together, these results suggest that higher psychopathic traits in incarcerated females are associated with shifts in network dynamics associated with perspective-taking, rather than blunted regional responses within the salience network. Additionally, this study highlights the importance of considering independent contributions of different dimensions of psychopathy.

Across the full sample of participants, perceiving painful compared to non-painful stimuli elicited the expected increased hemodynamic response across dACC, aINS, IFG, TPJ, and SMA (Fig. 2, Table S1 and S3), which is consistent with previous investigations of empathy for pain (Christov-Moore and Iacoboni, 2019, Lamm et al., 2011). Moreover, there was a large overlap between clusters responsive to painful situations during both imagine-self and imagine-other blocks, including bilateral pSTS/TPJ, dACC/SMA, and IFG/aINS. In particular, the hemodynamic response in dACC and aINS replicates multivariate analyses demonstrating overlap between voxel representations of experienced and vicarious pain (Corradi-Dell’Acqua et al., 2016). A previous study using the same task in male inmates found a positive association between psychopathic traits and response in aINS and dACC, particularly for the imagine-self blocks (Decety et al., 2013a). While no positive relationships between psychopathic traits and hemodynamic response was observed in this sample, the fact that there were no significant effects of psychopathy scores observed here is consistent with the notion that sensorimotor processing is intact in psychopathy (Espinoza et al., 2018).

Females with higher interpersonal and affective psychopathic traits exhibited decreased functional connectivity between rTPJ and pSTS during the perception of pain in others (Fig. 4). This is consistent with reduced integration between observed actions and inferred mental state (Schurz et al., 2021), as well as decreased attention towards physical pain (Yoder et al., 2015a). In contrast, higher Factor 2 scores were associated with stronger connectivity between raINS and PCC/precuneus during pain perception. The precuneus plays an important role across different types of perspective-taking (Schurz et al., 2021), and this positive association suggests that women with higher Factor 2 scores required more effort to integrate the saliency of pain signals encoded by aINS with third-person representations. These interpretations should be further investigated using more diverse stimuli such as dynamic depictions of other kinds of goal-directed actions.

The perspective-taking manipulation revealed that imaging oneself, compared to imagining another person in pain, was associated with increased activation in somatomotor cortices, dACC, and right IFG (Fig. 2A). This egocentric bias is consistent with previous work demonstrating that first-person perspectives elicit greater engagement of sensorimotor systems (Jackson et al., 2006, Ruby and Decety, 2001). In contrast, imagine-other blocks elicited greater response in extrastriate body area and the most posterior aspects of the TPJ, which was predicted given the role these regions play in perspective taking (Schurz et al., 2014).

During imagine-other blocks, but not imagine-self blocks, there was a large cluster in precuneus and more widespread activation in dmPFC. This result conceptually replicates previous work with incarcerated males in whom activation in these regions was greater when making trait attribution judgments for another person compared to judgements for oneself (Deming et al., 2018). The involvement of precuneus exclusively in the imagine-other block is also consistent with a large body of work highlighting the role of precuneus and PCC in perspective-taking (Healey and Grossman, 2018, Schurz et al., 2021, 2013).

Interestingly, the current study found no evidence that psychopathic traits affected connectivity from the core nodes of the salience network during perspective-taking. Instead, scores for Factor 1 and Factor 2 were associated with different changes in network dynamics within nodes of the social cognition network, specifically TPJ and amygdala (Fig. 5). Interestingly, for each seed, Factor 1 scores were associated with increased connectivity (stronger bias towards imagine-other), while Factor 2 scores led to reduced effective connectivity (stronger bias towards imagine-self). When viewing somatic pain from a third-person perspective and attempting to imagine being in that situation, individuals with higher Factor 1 scores demonstrated increased connectivity from social cognition nodes to sensorimotor regions. This suggests that female inmates with higher Factor 1 scores were less likely to experience the negative affect normally associated with witnessing others in pain, and so were more likely to engage in motor resonance (Buckholtz and Marois, 2012, Decety and Cowell, 2018). However, future work will be needed to clarify this effect, potentially by obtaining subjective pain perception ratings.

Factor 2 scores, which capture impulsive-antisocial behaviors, predicted reduced connectivity from left amygdala to left TPJ, right amygdala to precuneus, and left TPJ to right TPJ and dACC/SMA. TPJ and precuneus are crucial for mental state attributions (Schurz et al., 2021, Schurz et al., 2015), so these results suggest that when female inmates with higher impulsive-antisocial traits are asked to imagine witnessing another person in pain, they are less likely to attribute mental states to that person. This interpretation is bolstered by recent evidence that the TPJ is crucial for supporting spontaneous mental state attributions (Boccadoro et al., 2019). Moreover, reduced connectivity with dACC/SMA aligns with prior studies suggesting that higher psychopathic traits lead individuals to encode the pain and distress of others as less salient (Decety et al., 2013a, Yoder et al., 2015b, Yoder et al., 2015a). Previous research demonstrates that explicitly cuing participants to “feel with” others helps normalize brain responses in psychopathy (Meffert et al., 2013). However, in this study participants were merely asked to imagine the limb belonged to another person, without any instruction to engage in theory of mind. Given the association with Factor 2 instead of Factor 1, this effect might also reflect previous experience inflicting harm, rather than an interpersonal or affective effect. Future work could investigate this empirically by carefully accounting for previous violent behavior.

There are several limitations of the current study that future work could improve upon. There were no direct measures of prosocial or antisocial behavior, so it is not possible to empirically test whether the observed relationships between psychopathic traits and functional connectivity translate to differences in behaviors outside the scanner that might be motivated by empathy. Finally, the stimuli here depicted hands and feet in pain (or not), so our results may not generalize across other kinds of harmful situations, such as interpersonal harm or emotional abuse.

## 5 Conclusion

Overall, the results of the current study extend previous functional neuroimaging work identifying associations between psychopathic personality traits and alterations in functional connectivity from core nodes of the salience and social cognition networks. Female inmates with higher levels of lifestyle and antisocial personality traits demonstrated diminished neuronal coupling between nodes of the salience and social cognition networks. In contrast, variation in interpersonal and affective traits was associated with stronger connectivity to sensorimotor regions. These findings contribute to a growing body of evidence that Factor 2 and Factor 1 play distinct roles in female psychopathy, and that sex is an important variable in forensic neuroscience.

### CRediT authorship contribution statement

**Keith J. Yoder:** Formal analysis, Methodology, Software, Writing – original draft, Writing – review & editing, Visualization. **Carla L. Harenski:** Data curation, Validation, Writing – original draft. **Kent A. Kiehl:** Data curation, Project administration, Writing – original draft. **Jean Decety:** Conceptualization, Methodology, Writing – original draft, Writing – review & editing, Funding acquisition.

## Declaration of Competing Interest

The authors declare that they have no known competing financial interests or personal relationships that could have appeared to influence the work reported in this paper.
