# Supplementary material for: Psychopathic traits modulate functional connectivity during pain perception and perspective-taking in female inmates
Source: Neuroimage Clin. 2022 Mar 5;34:102984. doi: 10.1016/j.nicl.2022.102984 (PMC8907686; doi:10.1016/j.nicl.2022.102984)
Supplement: Supplementary data 1 [file mmc1.docx]

Table S1. Whole-brain results for pain perspective-taking task.

|  |  | MNI Coordinates | | |  |  |
| --- | --- | --- | --- | --- | --- | --- |
| Condition | Region | X | Y | Z | k | T |
| Pain > No-Pain | |  |  |  |  |  |
|  | L Inferior Frontal | -42 | 17 | -5 | 4963 | 10.46 |
|  | dACC | -3 | 23 | 31 |  | 9.00 |
|  | SMA | -6 | -4 | 61 |  | 6.09 |
|  | dlPFC | -30 | 44 | 28 |  | 7.15 |
|  | Precuneus | -9 | -73 | 37 |  | 5.31 |
|  | R Inferior Frontal | 48 | 11 | 1 | 1236 | 9.06 |
|  | R amygdala | 21 | 2 | -20 |  | 5.33 |
|  | R striatum | 21 | 8 | 7 |  | 5.09 |
|  | L TPJ | -60 | -43 | 28 | 736 | 8.50 |
|  | R TPJ | 60 | -31 | 25 | 385 | 7.48 |
|  | R Cerebellum | 36 | -58 | -32 | 254 | 6.18 |
|  | L Cerebellum | -30 | -64 | -29 | 99 | 5.15 |
|  | R Middle Temporal | 54 | -34 | -5 | 69 | 4.91 |
|  | Thalamus | -9 | -19 | 7 | 101 | 4.48 |
|  | L Superior Parietal | -24 | -70 | 49 | 102 | -4.19 |
|  | L Superior Frontal | -27 | -7 | 61 | 76 | -4.44 |
|  | L Occipital | -12 | -91 | -5 | 126 | -5.04 |
|  | R Superior Temporal | 63 | -10 | -5 | 98 | -6.06 |
|  | R Middle Frontal | 30 | 8 | 55 | 251 | -6.24 |
|  | R Occipital | 18 | -97 | 16 | 1190 | -6.29 |
| Self > Other | |  |  |  |  |  |
|  | R Insula | 45 | 8 | -8 | 350 | 5.12 |
|  | R Fusiform | 33 | -64 | -14 | 62 | -3.82 |
|  | R Occipital | 45 | -73 | 1 | 331 | -4.76 |
|  | L Occipital | -33 | -82 | 22 | 87 | -3.96 |
|  | SFG | -15 | -7 | 73 | 1467 | 6.47 |
|  | SMA | 3 | -10 | 58 |  | 4.39 |
|  | dACC | 6 | 11 | 46 |  | 3.95 |
| Perspective x Pain | |  |  |  |  |  |
|  | Cerebellum | 6 | -40 | -11 | 129 | 4.26 |
|  | R Insula | 51 | -10 | 4 | 118 | 4.35 |
| Clusters significant at FWEp < .05 (threshold p=.001, extent k=60). dACC: dorsal anterior cingulate cortex; SMA: supplementary motor area; dlPFC; dorsolateral prefrontal cortex; TPJ: temporoparietal junction; SFG: superior frontal gyrus. | | | | | | |

Table S2. Whole-brain results for pain empathy within each perspective

|  |  | MNI Coordinates | | |  |  |
| --- | --- | --- | --- | --- | --- | --- |
| Condition | Region | X | Y | Z | k | T |
| Imagine-self: Pain-No Pain | |  |  |  |  |  |
|  | L IFG | -54 | 11 | -2 | 3087 | 8.82 |
|  | SMA | 6 | 2 | 64 |  | 7.77 |
|  | dACC | -3 | 20 | 31 |  | 6.31 |
|  | R IFG | 54 | 8 | 4 | 839 | 7.58 |
|  | R Amygdala | 21 | 2 | -20 |  | 4.81 |
|  | L TPJ | -57 | -28 | 22 | 526 | 6.97 |
|  | R TPJ | 57 | -31 | 25 | 339 | 6.58 |
|  | R Cerebellum | 33 | -58 | -32 | 303 | 5.10 |
|  | Cerebellum | 3 | -64 | -17 | 105 | 4.86 |
|  | L Cerebellum | -33 | -61 | -29 | 81 | 4.76 |
|  | R Middle Temporal | 54 | -34 | -5 | 90 | 4.68 |
|  | Thalamus | -3 | -22 | 4 | 167 | 4.32 |
|  | R Middle Frontal | 30 | 5 | 55 | 134 | -5.01 |
|  | R Occipital | 15 | -85 | -11 | 155 | -5.29 |
|  | R Superior Parietal | 30 | -67 | 43 | 519 | -5.86 |
| Imagine-other: Pain-No Pain | |  |  |  |  |  |
|  | L Insula | -39 | 17 | -8 | 3322 | 8.59 |
|  | dACC | -3 | 23 | 31 |  | 7.32 |
|  | dlPFC | -27 | 47 | 22 |  | 5.50 |
|  | precuneus | -6 | -70 | 34 |  | 5.57 |
|  | R Insula | 42 | 26 | -2 | 564 | 6.90 |
|  | L TPJ | -63 | -34 | 25 | 488 | 5.95 |
|  | R TPJ | 63 | -28 | 25 | 163 | 5.65 |
|  | R Postcentral | 42 | -28 | 58 | 103 | -3.96 |
|  | Cerebellum | 0 | -49 | -5 | 66 | -4.05 |
|  | L Postcentral | -39 | -31 | 64 | 300 | -4.27 |
|  | L Middle Temporal | -54 | -16 | -2 | 64 | -4.58 |
|  | R Middle Frontal | 30 | 11 | 58 | 95 | -4.67 |
|  | R Postcentral | 18 | -37 | 73 | 276 | -4.82 |
|  | R Superior Temporal | 63 | -10 | -5 | 191 | -6.38 |
|  | Occipital | 18 | -97 | 16 | 248 | -6.62 |
| Clusters significant at FWEp < .05 (threshold p=.001, extent k=60). SMA: supplementary motor area; dACC: dorsal anterior cingulate cortex; IFG: inferior frontal gyrus; TPJ: temporoparietal junction; dlPFC: dorsolateral prefrontal cortex. | | | | | | |

Table S3. Functional connectivity for pain vs. no-pain

|  |  | MNI Coordinates | | |  |  |
| --- | --- | --- | --- | --- | --- | --- |
| Seed | Region | X | Y | Z | k | T |
| dACC |  |  |  |  |  |  |
|  | R Fusiform | 42 | -46 | -26 | 84 | 5.03 |
|  | R Precentral | 30 | -7 | 58 | 1606 | 5.00 |
|  | Paracentral Lobule | -12 | -22 | 70 |  | 4.65 |
|  | SMA | 9 | 5 | 49 |  | 4.31 |
|  | Cuneus | 12 | -79 | 4 | 71 | 4.76 |
|  | L Insula | -45 | -13 | -2 | 73 | 4.22 |
|  | L Temporal Pole | -51 | 11 | -14 | 94 | 4.22 |
|  | R Occipital | 33 | -76 | 25 | 98 | 4.21 |
|  | R Insula | 42 | -22 | 10 | 103 | 4.11 |
|  | R Precentral | 54 | -4 | 37 | 124 | 4.04 |
| R aINS |  |  |  |  |  |  |
|  | R Precentral | 63 | 2 | 16 | 102 | 5.01 |
|  | Paracentral Lobule | 15 | -31 | 67 | 95 | 4.55 |
| R TPJ |  |  |  |  |  |  |
|  | Paracentral Lobule | 0 | -28 | 67 | 318 | 4.51 |
|  | SMA | 9 | -4 | 70 |  | 4.08 |
|  | R Postcentral | 51 | -10 | 34 | 171 | 4.45 |
|  | L Precentral | -39 | -16 | 55 | 143 | 4.41 |
|  | L Superior Temporal | -54 | -19 | 4 | 88 | 4.20 |
|  | Superior Frontal | -6 | 59 | 31 | 167 | 4.13 |
| L TPJ |  |  |  |  |  |  |
|  | L Postcentral | -54 | -10 | 34 | 489 | 5.77 |
|  | L Temporal Pole | -54 | -1 | -23 |  | 4.06 |
|  | R Postcentral | 54 | -13 | 28 | 70 | 4.14 |
| No significant clusters identified for left insula or amygdala seeds. Clusters significant at FWEp < .05 (threshold p=.001, extent k=60). dACC: dorsal anterior cingulate cortex; aINS: anterior insula; TPJ: temporoparietal junction; SMA: supplementary motor area | | | | | | |

Table S4. Psychopathy and changes in functional connectivity for pain vs no-pain

|  |  |  | MNI Coordinates | | |  |  |
| --- | --- | --- | --- | --- | --- | --- | --- |
| Seed | Factor | Region | X | Y | Z | k | T |
| R aINS | |  |  |  |  |  |  |
|  | Factor 2 |  |  |  |  |  |  |
|  |  | PCC | 0 | -52 | 31 | 255 | 4.22 |
| R TPJ | |  |  |  |  |  |  |
|  | Factor 1 |  |  |  |  |  |  |
|  |  | R pSTS | 60 | -34 | 7 | 78 | -5.01 |
| Clusters significant at FWEp < .05 (threshold p=.001, extent k=60). aINS: anterior insula; PCC: posterior cingulate cortex; TPJ: temporoparietal junction; pSTS: posterior superior temporal sulcus | | | | | | | |

Table S5. Functional connectivity results for imagine-self vs imagine-other

|  |  | MNI Coordinates | | |  |  |
| --- | --- | --- | --- | --- | --- | --- |
| Seed | Region | X | Y | Z | k | T |
| R aINS |  |  |  |  |  |  |
|  | L Temporal Pole | -42 | 11 | -20 | 309 | -6.58 |
|  | dmPFC | 0 | 59 | 34 | 4471 | -6.54 |
|  | R Temporal Pole | 45 | 2 | -11 | 320 | -7.07 |
|  | R IFG | 60 | 14 | 28 | 113 | -5.03 |
|  | L IFG | -54 | 11 | 28 | 66 | -4.31 |
| L aINS |  |  |  |  |  |  |
|  | L Temporal Pole | -42 | 11 | -20 | 283 | -7.50 |
|  | dmPFC | -6 | 44 | 52 | 3798 | -6.87 |
|  | R Temporal Pole | 45 | 11 | -17 | 223 | 5.90 |
| dACC |  |  |  |  |  |  |
|  | dmPFC | 0 | 50 | 43 | 5742 | -9.21 |
|  | R Temporal Pole | 45 | 5 | -11 | 389 | -8.02 |
|  | L Temporal Pole | -42 | 11 | -20 | 344 | -8.02 |
| R TPJ |  |  |  |  |  |  |
|  | R Insula | 48 | 2 | -11 | 410 | -7.93 |
|  | mPFC | 0 | 59 | 34 | 4703 | -7.64 |
|  | L Insula | -42 | 8 | -20 | 287 | -7.17 |
|  | L dlPFC | -54 | 17 | 25 | 62 | -4.11 |
| L TPJ |  |  |  |  |  |  |
|  | L Temporal Pole | -42 | 8 | -20 | 455 | -7.45 |
|  | R Temporal Pole | 45 | 11 | -17 | 180 | -7.26 |
|  | dmPFC | 3 | 53 | 40 | 2939 | -7.43 |
|  | R IFG | 60 | 17 | 28 | 129 | -5.07 |
|  | L IFG | -54 | 11 | 31 | 87 | -4.62 |
| R Amygdala |  |  |  |  |  |  |
|  | R Insula | 45 | 11 | -20 | 223 | -6.04 |
|  | L Insula | -24 | 8 | -14 | 145 | -5.72 |
|  | dACC/SMA | 0 | 26 | 46 | 1650 | -5.33 |
|  | L IPL | -63 | -40 | 25 | 101 | -4.40 |
| L Amygdala |  |  |  |  |  |  |
|  | dmPFC | 3 | 56 | 31 | 847 | -5.03 |
|  | SMA | 15 | 14 | 64 |  | -4.78 |
|  | L Temporal Pole | -39 | 11 | -20 | 136 | -4.88 |
| Clusters significant at FWEp < .05 (threshold p=.001, extent k=60). aINS: anterior insula; dACC: dorsal anterior cingulate cortex; dmPFC: dorsomedial prefrontal cortex; IFG: inferior frontal gyrus; SMA: supplementary motor area; IPL: inferior parietal lobule. | | | | | | |

Table S6. Psychopathy and changes in functional connectivity for imagine-other vs imagine-self

|  |  |  | MNI Coordinates | | |  |  |
| --- | --- | --- | --- | --- | --- | --- | --- |
| Seed | Factor | Region | X | Y | Z | k | T |
| R TPJ | |  |  |  |  |  |  |
|  | Factor 1 |  |  |  |  |  |  |
|  |  | L Postcentral | -51 | -31 | 49 | 140 | -4.74 |
|  | Factor 2 |  |  |  |  |  |  |
|  |  | R dlPFC | 48 | 26 | 28 | 200 | 4.48 |
| L TPJ | |  |  |  |  |  |  |
|  | Factor 1 |  |  |  |  |  |  |
|  |  | R Temporal Pole | 39 | 5 | -20 | 239 | 5.55 |
|  |  | L Temporal Pole | -39 | 29 | 1 | 87 | 5.38 |
|  |  | Precuneus | 12 | -55 | 49 | 96 | 4.47 |
|  |  | L Insula | -30 | 8 | -17 | 70 | 4.43 |
|  |  | Cuneus | 15 | -73 | 10 | 65 | 4.35 |
|  |  | R Middle Temporal gyrus | 57 | -49 | 1 | 164 | 3.93 |
|  | Factor 2 |  |  |  |  |  |  |
|  |  | L Insula | -30 | 11 | -5 | 65 | -5.17 |
|  |  | L aINS | -33 | 32 | 1 | 60 | -5.03 |
|  |  | dACC | 12 | 26 | 28 | 211 | -4.94 |
|  |  | Midbrain | -9 | -16 | -20 | 80 | -4.17 |
|  |  | R Middle Temporal gyrus | 57 | -58 | 10 | 74 | -4.15 |
| R Amygdala | |  |  |  |  |  |  |
|  | Factor 1 |  |  |  |  |  |  |
|  |  | Operculum | 57 | -4 | 4 | 83 | -4.12 |
|  |  | L STS | -57 | -10 | 10 | 191 | -4.68 |
|  |  | Midcingulate | -6 | -19 | 34 | 89 | -5.07 |
|  |  | L Postcentral | -45 | -28 | 49 | 369 | -5.36 |
|  |  | R Precentral | 27 | -22 | 64 | 106 | -4.53 |
|  | Factor 2 |  |  |  |  |  |  |
|  |  | Precuneus | -6 | -49 | 43 | 64 | 4.27 |
| L Amygdala | |  |  |  |  |  |  |
|  | Factor 2 |  |  |  |  |  |  |
|  |  | L TPJ | -54 | -55 | 31 | 186 | 4.87 |
|  |  | Precuneus | 21 | -46 | 46 | 77 | 4.26 |
|  |  | L dlPFC | -39 | 11 | 43 | 69 | 4.31 |
| Clusters significant at FWEp < .05 (threshold p=.001, extent k=60). TPJ: temporoparietal junction; dlPFC: dorsolateral prefrontal cortex; aINS: anterior insula; dACC: dorsal anterior cingulate cortex; STS: superior temporal sulcus | | | | | | | |

Table S7. Regions and functions potentially impacted by psychopathy

| Region | Function(s) |
| --- | --- |
| Amygdala | saliency, affective processing, valuation |
| Anterior Insula (aINS) | saliency, affective processing, somatosensation |
| Cerebellum | motor coordination |
| Cuneus | sensory processing |
| Dorsal Anterior Cingulate (dACC) | saliency, affective processing |
| Dorsolateral Prefrontal Cortex (dlPFC) | attention |
| Dorsomedial Prefrontal Cortex (dmPFC) | social cognition |
| Fusiform | sensory processing, social cognition |
| Inferior Frontal Gyrus (IFG) | action observation, somatosensation |
| Inferior Parietal Lobule (IPL) | social cognition, attention |
| Insula | somatosensation |
| Medial Prefrontal Cortex (mPFC) | social cognition, valuation |
| Midbrain | motivation |
| Midcingulate | somatosensation |
| Middle Frontal | action observation, attention |
| Middle Temporal | action observation |
| Occipital Cortex | sensory processing |
| Operculum | somatosensation, saliency |
| Paracentral Lobule | social cognition |
| Postcentral Gyrus/Sulcus | sensorimotor processing |
| Posterior Cingulate Cortex (PCC) | social cognition |
| Posterior STS (pSTS) | social cognition, action observation |
| Precentral Gyrus/Sulcus | sensorimotor processing |
| Precuneus | social cognition |
| Striatum | valuation |
| Superior Frontal Gyrus (SFG) | attention |
| Superior Parietal Gyrus/Sulcus | social cognition, sensory integration |
| Superior Temporal Sulcus (STS) | social cognition, sensory integration |
| Supplementary Motor Area (SMA) | saliency, motor preparation |
| Temporal Pole | semantic representation, affective processing |
| Temporo-Parietal Junction (TPJ) | social cognition, sensory integration, attention |
| Thalamus | sensory integration |
| Deming & Koenigs, 2020; Fallon, Roberts, & Stancak, 2020; Schurz et al., 2021; Yoder & Decety, 2018; Neurosynth.org | |


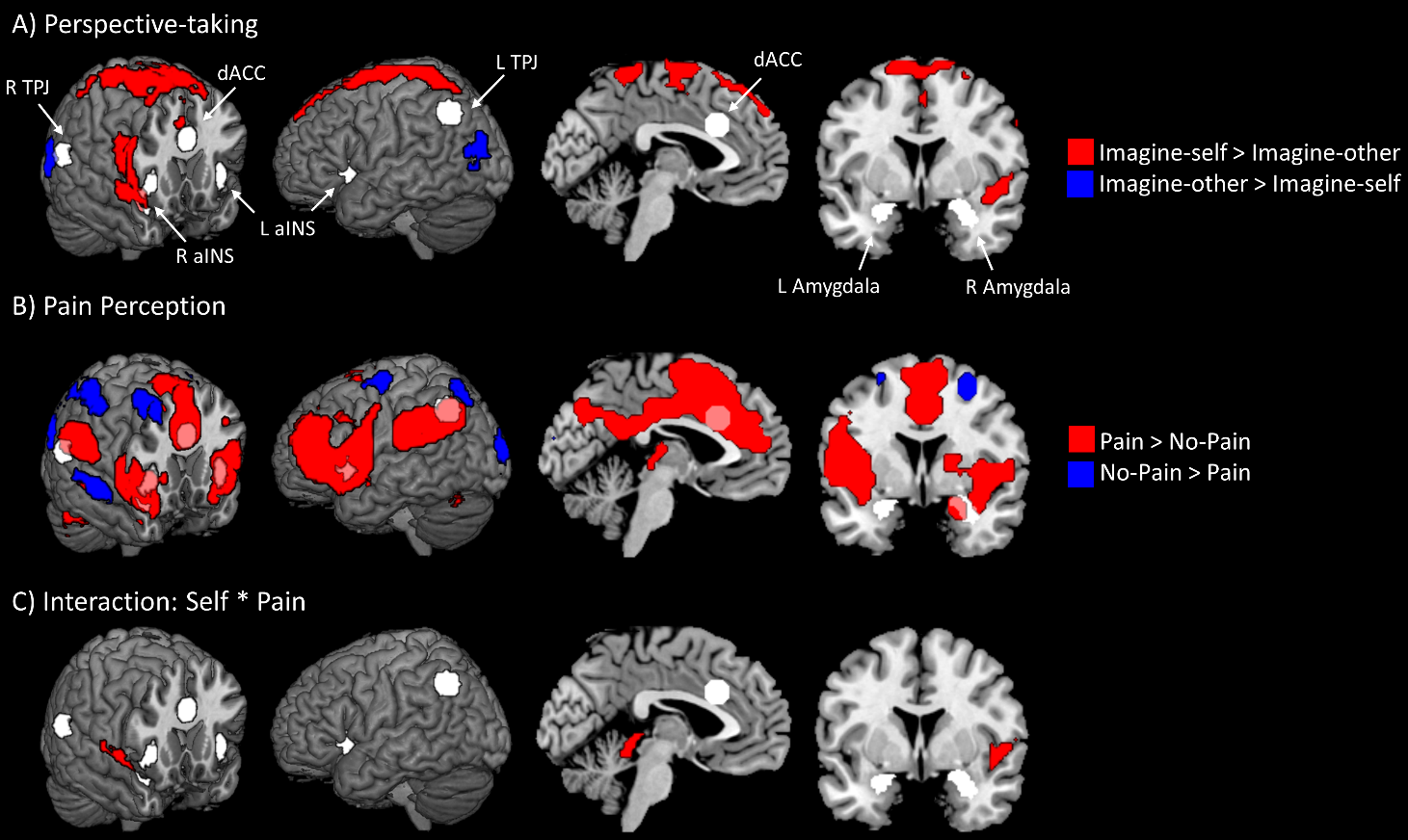


**Figure S1. Whole-brain main effects and interaction**. Significant clusters for the main effect of block perspective-taking instructions (A), main effect of pain depicted in stimuli (B), or the Self by Pain interaction (C). All clusters significant at family-wise error < .05 (height p=.001, extent k=60). A priori regions of interest used as seeds in the functional connectivity analysis are shown in white. dACC: dorsal anterior cingulate cortex; R aINS: right anterior insula cortex; L aINS: left anterior insula cortex; R TPJ: right temporoparietal junction; L TPJ: left temporoparietal junction
